# The efficacy and safety of Xuebijing injection as an adjunctive treatment for acute pancreatitis: Protocol for a systematic review and meta-analysis of randomized controlled trials

**DOI:** 10.1097/MD.0000000000018743

**Published:** 2020-01-24

**Authors:** Qilin Tang, Lixin Tian, Chao Gao, Kai Zhang, Nan Su, Baohong Liu, Jingbo Zhai, Si Liu, Yan Li

**Affiliations:** a School of Basic Medical Sciences, Hebei University of Chinese Medicine, Hebei, 050200; b School of Acupuncture-Moxibustion and Tuina, Tianjin University of Traditional Chinese Medicine, Tianjin, 301617; c Department of General Surgery, Tianjin General Surgery Institute, Tianjin Medical University General Hospital, Tianjin, 300052; d Department of Acupuncture and Moxibustion, Tianjin Gong An Hospital, Tianjin, 300042; e Institute of Traditional Chinese Medicine, Tianjin University of Traditional Chinese Medicine, Tianjin, 301617; f Tianjin Chase Sun Pharmaceutical Co., Ltd, Tianjin, 301700, China.

**Keywords:** acute pancreatitis, protocol, randomized controlled trials, systematic review, Xuebijing injection

## Abstract

**Background:**

Acute pancreatitis (AP) is one of the common diseases with increasing incidence in clinical surgery and other gastrointestinal-digestive departments. Despite the rapid development of modern medicine, the overall mortality rate of AP is still high. Xuebijing (XBJ) injection (a traditional Chinese patent medicine) is a potentially effective drug for AP. This study is designed to assess the efficacy and safety of XBJ injection for AP.

**Methods:**

We will extract data and assess methodological quality of included studies from 7 electronic databases from their inception to December 31, 2019. The primary outcomes include the mortality, surgical intervention, systemic inflammatory response syndrome (SIRS), local complications, systemic infections, gastrointestinal symptoms, and normal blood amylase recovery time. The statistical analysis will be performed using RevMan 5.3 software.

**Results:**

This study will provide high-quality evidence for the efficacy of XBJ injection as an adjuvant therapy for AP.

**Conclusion:**

The study will provide the key evidence for clinical doctors and the development of clinical guidelines.

## 1 Introduction

Acute pancreatitis (AP) is a potentially fatal disease that can cause patients to enter the emergency room or intensive care unit.^[1–3]^ Despite the rapid development of modern medicine and the discovery of a large number of prognostic markers and predictors of inflammation, the total mortality of AP is still very high.^[4,5]^ In addition, anti-inflammatory treatment of acute pancreatitis, including prophylactic antibiotics, remains controversial.^[6–8]^

With the rapid and extensive development of the treatment of AP with the combination of traditional Chinese and Western medicine, the incidence and mortality of sepsis, abdominal compartment syndrome, and other complications of AP have been effectively reduced.^[9–12]^ At present, Xuebijing (XBJ) injection, a mixture of 5 Chinese herbs, has been proved to protect vascular endothelial cells, improve microcirculation and tissue perfusion, regulate immunity, attenuate acute organ injury and dysfunction, and relieve epigastric pain.^[13–15]^ XBJ injection has treatment effects on sepsis and multiple organ dysfunction syndrome (MODS).^[13,16]^ It has the anti-inflammatory effect by reducing the expression of inflammatory factors such as Toll-like receptor-4 and NF-KB.^[16]^ So it has been paid more attention to the treatment of other critical diseases. Some researchers have used it to treat AP and have achieved convincing evidence.^[17,18]^ With new published high-quality researches, it is important to evaluate the efficacy of XBJ injection for AP. Therefore, our aim is to systematically evaluate the efficacy of XBJ injection for AP.

## 2 Methods

This protocol will follow the Preferred Reporting Items for Systematic Review and Meta-analysis Protocols (PRISMA-P).

### 2.1 Criteria for inclusion in the study

#### 2.1.1 Types of studies

Randomized controlled trials (RCTs) will be included. We will exclude quasi-randomized controlled trials (quasi-RCTs) with non-random methods, such as date of admission, date of birth, or clinic record number. The language will be unlimited.

#### 2.1.2 Types of participants

Patients with AP (age ≥18 years) will be included. There is no restriction on gender. AP is diagnosed based on internationally recognized diagnostic standards.^[19–21]^ Patients with AP should be hospitalized within 48 hours.

#### 2.1.3 Types of interventions

The intervention is XBJ plus routine treatment in the treatment group and only routine treatment in the control group. The routine treatment includes fluid resuscitation, antibiotic therapy, nutritional support, or mechanical ventilation.

#### 2.1.4 Types of outcomes

The primary outcomes include mortality, surgical intervention, systemic inflammatory response syndrome, local complications, systemic infection (septicemia, urinary tract infection, and pneumonia), gastrointestinal symptoms (the relief time of abdominal pain, bloating relief time, anal exhaust recovery time, defecation recovery time, bowel sound recovery time), and normal blood amylase recovery time.

Secondary outcomes include Acute Physiology and Chronic Health Evaluation II score, hospitalization time, inflammatory markers (such as C-reactive protein), and adverse events.

### 2.2 Search strategy

Two reviewers (CG and KZ) will independently search PubMed, Web of Science, EMBASE, Cochrane Central Register of Controlled Trials, Wan Fang Data, Chinese Scientific Journal Database, and China National Knowledge Infrastructure (CNKI) from their inception to December 31, 2019. Table 1 shows the search strategy for PubMed. ClinicalTrials.gov will also be searched to identify potentially eligible studies.

**Table 1:**
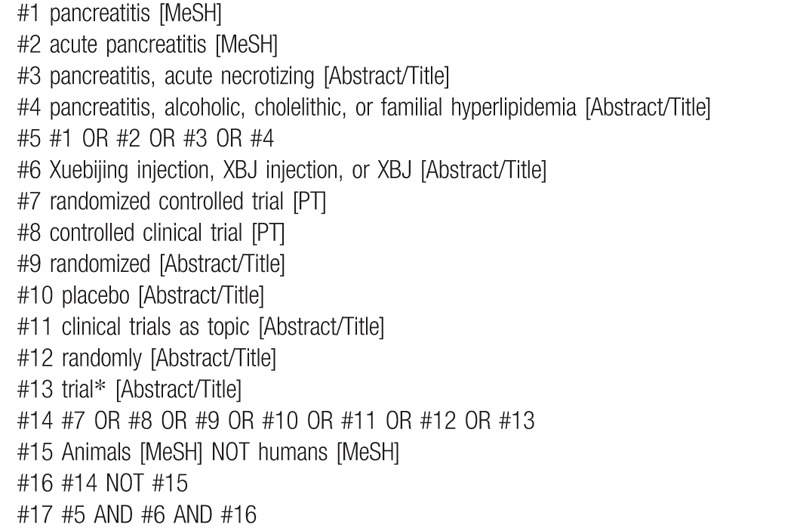
Search strategy for PubMed.

### 2.3 Selection of studies

The titles and abstracts will be checked to exclude irrelevant papers. Then, filter the remaining studies by reading the full texts. Two authors will cross-check results, discuss, and resolve the disagreement.

### 2.4 Data extraction and management

Two reviewers (BHL and NS) will extract the relevant data independently. Any difference will be evaluated by a third reviewer (KZ). The information such as study design, characteristics of patients, interventions, and outcomes will be extracted. If the data is incomplete, we will try to contact the first author or corresponding author for obtaining the missed information.

### 2.5 Risk of bias

Two authors (CG and KZ) will independently investigate the risk of bias for each included study with a tool in the Cochrane handbook. It will be graded as low, unclear, or high. Disagreements will be resolved by discussion or consultation with another author (QLT).

### 2.6 Measures of treatment effect

We will conduct statistical analysis by RevMan 5.3 software. Risk ratio (RR) will be used to estimate the effect for the dichotomous variables. Mean difference (MD) will be used to represent the estimate of the effect for continuous variables. We will also report the 95% confidence intervals (CIs). When the same result is measured in a variety of ways, standard mean difference (SMD) will be used to describe the intervention effect.

### 2.7 Assessment of heterogeneity

The *I*^2^ value will be computed to quantify the statistical heterogeneity in the meta-analysis. The *I*^2^ value will be divided into 4 categories:

(1) 0% to 40%;
(2) 30% to 60%;
(3) 50% to 90%;
(4) 75% to 100%

### 2.8 Data synthesis

Heterogeneity test will be conducted before conducting the meta-analysis. If *I*^2^ < 50% and *P* > .10, the heterogeneity will be low. The fixed-effects model will be used to estimate the effect. On the contrary, the random-effects model will be adopted. Descriptive analysis will be performed when clinical heterogeneity is not neglected. Publication bias will be graphically examined by the funnel plot when the meta-analysis contains 10 or more studies.^[22]^ We will conduct subgroup analysis according to mild and severe AP, age, race, and drug dosage.^[19,23–25]^ The robustness of the meta-analysis will be tested based on risk of bias and sample size by sensitivity analysis.

### 2.9 Assessment of evidence quality

We will investigate the quality of evidence for all outcomes by the Grading of Recommendations Assessment, Development and Evaluation (GRADE) tool. The level of evidence is divided into 4 types (very low, low, medium, or high).

### 2.10 Ethics and dissemination

There is no need for ethical approval because individual information cannot be identified. The findings and important protocol amendments will be reported in a peer-reviewed journal.

## 3 Discussion

This study will provide the high-quality evidence on XBJ injection for acute pancreatitis, and a reference for clinical doctors and the development of clinical guidelines. Some potential limitations may affect the conclusions drawn from the study. First, different drug doses, the severity of acute pancreatitis may increase the risk of heterogeneity. Second, it is difficult to adopt the blinding in RCTs about traditional Chinese medicine injections for AP.^[26–30]^ We need to explain results with caution.

## Author contributions

YL conceived the study and provided general guidance for the drafting of the protocol. CG and KZ drafted the protocol. QLT, LXT, and KZ designed the search strategy. QLT, LXT, CG, KZ, NS, BHL, JBZ, and SL drafted the manuscript. QLT, LXT, CG, KZ, NS, BHL, JBZ, SL, and YL reviewed and revised the manuscript. All authors have read and approved the final version of the manuscript.
